# Under-reporting of major birth defects in Northwest Russia: a registry-based study

**DOI:** 10.1080/22423982.2017.1366785

**Published:** 2017-08-31

**Authors:** Anton A. Kovalenko, Tormod Brenn, Jon Øyvind Odland, Evert Nieboer, Alexandra Krettek, Erik Eik Anda

**Affiliations:** ^a^ Department of Community Medicine, UiT The Arctic University of Norway, Tromsø, Norway; ^b^ International School of Public Health, Northern State Medical University, Arkhangelsk, Russia; ^c^ Department of Biochemistry and Biomedical Sciences, McMaster University, Hamilton, Canada; ^d^ Department of Biomedicine and Public Health, School of Health and Education, University of Skövde, Skövde, Sweden; ^e^ Department of Internal Medicine and Clinical Nutrition, Institute of Medicine, Sahlgrenska Academy at University of Gothenburg, Gothenburg, Sweden

**Keywords:** Birth defect, birth registry, linkage, under-reporting

## Abstract

The objective was to assess the prevalence of selected major birth defects, based on data from two medical registries in Murmansk County, and compare the observed rates with those available for Norway and Arkhangelsk County, Northwest Russia. It included all newborns (≥22 completed weeks of gestation) registered in the Murmansk County Birth Registry (MCBR) and born between 1 January 2006 and 31 December 2009 (n=35,417). The infants were followed-up post-partum for 2 years through direct linkage to the Murmansk Regional Congenital Defects Registry (MRCDR). Birth defects identified and confirmed in both registries constituted the “cases” and corresponded to one or more of the 21 birth defect types reportable to health authorities in Moscow. The overall prevalence of major birth defects recorded in the MRCDR was 50/10,000 before linkage and 77/10,000 after linkage with the MCBR. Routine under-reporting to the MRCDR of 40% cases was evident. This study demonstrates that birth registry data improved case ascertainment and official prevalence assessments and reduced the potential of under-reporting by physicians. The direct linkage of the two registries revealed that hypospadias cases were the most prevalent among the major birth defects in Murmansk County.

**A** **bbreviations:** ICD-10, International Classification of Diseases, 10th revision; MCBR, Murmansk County Birth Registry; MRCDR, Murmansk Regional Congenital Defects Registry; MGC, Murmansk Genetics Center

## Background

Congenital anomalies (also known as birth defects) are structural or functional anomalies that exist at or before birth, although some become evident during infancy. Based on EUROCAT data, the total prevalence of all birth defects diagnosed at birth in Europe is about 2.5% [1] and its temporal prevalence is stable. Even so, congenital anomalies have become the main cause of perinatal mortality as other causes of death have declined [2]. Each year an estimated 7.9 million babies are born with serious birth defects and approximately 50% of all congenital malformations do not have an identified cause. Genetic factors, exposure to viruses or bacteria, maternal diseases and exposure to chemicals have been associated with increased risk [3]. Although some congenital birth defects are treatable (surgically or otherwise), annual estimates indicate that 3.2 million children are handicapped for life [4]. These children often need special medical treatment and may suffer from long-term effects, as well as socially [4]. Birth defects not only affect the child, but also the child’s family and society as a whole [5]. Because of the serious public health significance, understanding the causes of birth defects constitutes a growing priority, as do the development, implementation and evaluation of preventive programmes [6,7].

Acquisition of data from population-based registries of birth defects constitutes an important information source [8]. Since not all birth defects are detectable at delivery or even during the neonatal period, some defects, such as hearing defects or mental disorders, remain under-reported. Another deficiency is incomplete or incorrect recording by physicians [9].

The Murmansk County Birth Registry (MCBR) is based on the format used in the Nordic countries and was established in 2006. Pertinent information was systematically and routinely collected from the 15 county maternity clinics, each of which deliver 1–4 neonates per day (in total ~9000 deliveries annually). In 2010, it was the only operational birth registry in Russia [10]. The MCBR records information on birth defects in newly born babies with 22 completed weeks of gestation and diagnosed between birth and hospital discharge. In 1996, the Murmansk Regional Congenital Defects Registry (MRCDR) was established to collect information on all birth defects diagnosed in children from birth to 16 years of age. Mandatory reporting of 21 birth defect types to the National Birth Defects Surveillance Monitoring Programme has been in place since 1999. However, only 54 regions of 83 in Russia participated in this federal monitoring programme in 2011 [11]. There are several publications based on data from local Russian registries of birth defects. They focus on prevalence rates and time trends, but it is difficult to conduct a systematic scientific investigation (e.g. of case control design) of risk factors due to a lack of information in such registries [12,13]. In addition, there is no experience in Russia at the local or national level of linking such data with birth registries.

Recent studies demonstrate the effectiveness of using secondary databases to improve the quality of registry data [14]. One study in particular that combined hospital discharge data and cancer registry data reports that hospital discharge data added between 12% and 21% more cases [15]. In this context, we examined information from the MCBR and the MRCDR, with the overall objective of obtaining more reliable prevalence estimates of birth defects in Northwest Russia. To achieve this we (i) combined the results of these two registries; (ii) identified possible under-reporting; and (iii) compared the prevalences of birth defects in Murmansk County with those of Norway and Archangelsk County. The latter is located in the northern region of European Russia, and lies on the banks of the Northern Dvina River, near its exit into the White Sea.

## Materials and methods

According to the 2010 Census, Murmansk County in Northwest Russia had 795,409 inhabitants, with a population density of 6.2 per square kilometre [16,17]. The City of Murmansk is a port and the administrative centre of Murmansk County and is located not far from Russia’s borders with Norway and Finland. In 2010, the population of Murmansk City was 307,257 inhabitants [16]. Even though it has declined rapidly from 442,000 in 1989, it remains the largest city above the Arctic Circle. As already mentioned, the average annual number of deliveries in the region is around 9000. The study population consisted of all neonates registered in the MCBR between 1 January 2006 and 31 December 2009. Both singleton and multiple deliveries were included.

### The Murmansk county birth registry

We obtained detailed information on mothers and their newly born babies from the MCBR, as well as for birth defects diagnosed (included all livebirths, stillbirths and terminations) during the perinatal period, namely from ≥22 weeks of gestation to the hospital discharge 7–12 days post-partum, as appropriate for the type of delivery (normal or caesarean section) or any complications. The data in the MCBR derived from the mothers’ medical and obstetric records, the neonatal delivery records and from interviews with the mothers. The same physician or midwife who gathered the required information from medical and obstetric records conducted the interview and completed a two-page birth registry form comprised of 54 major fields of detailed medical and personal information about the mother and her baby/babies and father as well [10].

### The Murmansk regional congenital defects registry

**Figure 1.**
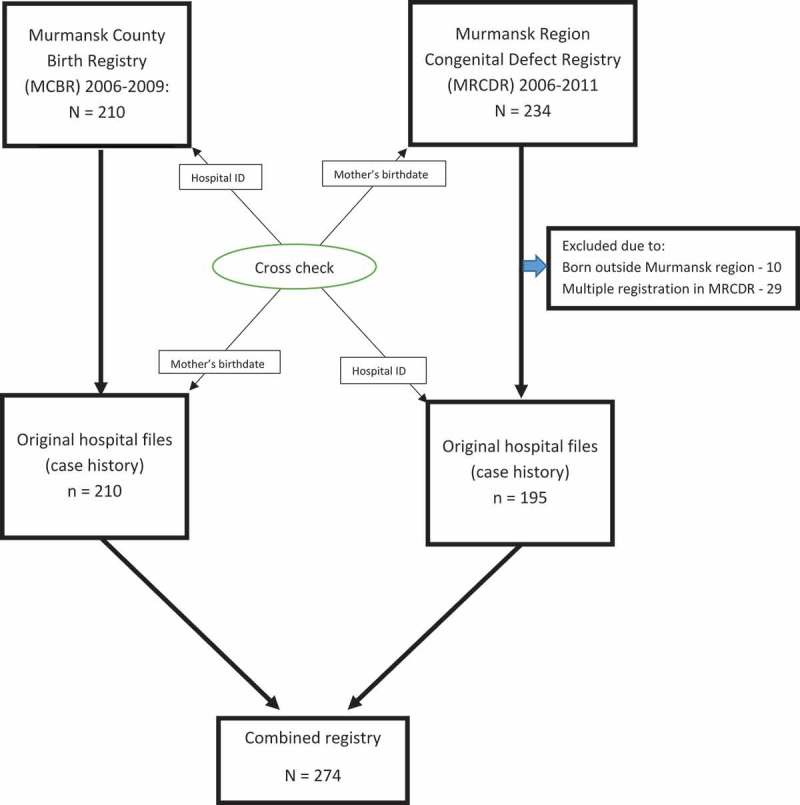
Number of major birth defect cases, exclusions and the manual linkage procedure of the Murmansk County Birth Registry (MCBR) and the Murmansk Regional Congenital Defects Registry (MRCDR). Note that, after the linkage procedure was completed, 64 new cases were added to the MCBR based on the MRCDR data and 79 to the latter from the former (includes cases up to age 2). Babies with multiple birth defects were excluded.

We extracted details about cases of major birth defects from the MRCDR, which included information on all birth defects diagnosed between birth (≥22 weeks of gestation and birth weight >500 grams) up to 2 years of age. The MCBR was a passive registry with its main sources of information being the maternity hospitals, children’s polyclinics (primary care), children’s hospitals and pathology departments and other medical institutions. On diagnosis of a birth defect, the physician completed a notice form and submitted it to the local Medical Analytic Information Centre for registration. The pertinent information was recorded in the MRCDR only after its confirmation by a medical institution. One exception were the notice forms issued by children’s polyclinics, which were exempt from the confirmation requirement. The MRCDR includes information on birth date, weight, vital status, whether multiple delivery, birth defect diagnosis, gender, gestational age, place of delivery, mother’s age, mother’s parity and mother’s place of residence at the time of delivery. Subsequently we selected all cases born within the study period 1 January 2006 to 31 December 2009. During the study period, 234 neonates registered in the MRCDR had major birth defects (see Figure 1). Of these, 17 cases were double entries, 6 triple and 10 were from outside of the Murmansk region; these cases were excluded automatically, leaving 195 children with major birth defects.

### Creation of a “combined registry”

For the linkage procedure, we selected all cases from the MRCDR with major birth defects for babies born between 1 January 2006 and 31 December 2009. The MRCDR electronic platforms changed during the study period from Medmonitor to Microsoft Excel, and subsequently to Microsoft Access; they were thereby fragmentised. We received only paper printouts from The Ministry of Health Care located in Murmansk City and, thus, the linking of the MCBR and the MRCDR was manual.

Based on place of delivery, date of birth of the mother and hospital ID file number in the MCBR, we requested all 210 original medical files from the maternity hospitals. Similarly, based on the same variables in the MRCDR, we requested 195 original medical files from maternity hospitals. After receiving these original files, we checked whether a case with a major birth defect had been in the MCBR, the MRCDR or in both. The 64 cases registered only in the MRCDR were combined with those in the MCBR using a manual (but direct) linkage algorithm, based on the original medical file and hospital ID number of the participant from the MCBR and the mother’s birthdate. Thus, the combined registry included 274 cases of major birth defects with the corresponding International Classification of Diseases, Revision 10 (ICD-10) code and date of diagnosis.

### Statistical analyses

We considered data on the 21 selected birth defects (referred to in this text as major birth defects), namely those included in the mandatory MRCDR annual report to the health authorities in Moscow. The statistical package SPSS version 21.0 (IBM Corp., 2012) was used to analyse and create descriptive statistics. We calculated confidence intervals based on the Wilson procedure, without correction for continuity.

Prevalence rates were calculated separately for the MCBR, MRCDR and the combined registry. Furthermore, we compared rates of major birth defects with those reported for Arkhangelsk County and Norway.

### Ethical considerations

The Regional Health Administration of Murmansk Oblast, as well as the Ethics Committee of the Association of Gynecologists & Obstetricians of Murmansk Oblast approved this study. The Regional Ethics Committee (REK) in Norway also granted ethical approval. After linkage, all data from the MCBR and the MRCDR were de-identified. Both registries (MCBR and MRCDR) were obligatory parts of the healthcare system in the Murmansk County during the study period. All study participants signed an agreement form kept in their hospital medical files about their willingness to share future observations and possible use of personal data for further research. Within the study period, no one declined to complete the written consent form.

## Results

Of the 35,417 neonates (live and stillborn) registered in the MCBR during the study period, 210 had major birth defects (see Figure 1).

The characteristics of the study population summarised in Table 1 reflect information obtained from both the MCBR and MRCDR; the latter did not yield any additional descriptive information about the mothers and children other than the birth defect diagnoses themselves. Among the 35,417 deliveries in the MCBR, 297 were multiple deliveries (0.8%). On average, maternal age was lower than paternal age at the time of delivery (26.5 and 29.5 years, respectively). At delivery, 81.2% of mothers were between 21 and 35 years of age. The average gestational age was 39.04 weeks, 3340 g was the average birth weight and 4109 women (11.7%) had previously experienced one or more spontaneous abortions. Multivitamin and folic acid intakes during pregnancy, respectively, were 91.3% and 70.7%, compared with 13.3% and 8.5% before pregnancy (Table 1).

**Table 1.** Characteristics of the study population.

Variables	n=35,417
Multiple deliveries (%)	297 (0.8)
Singleton deliveries	34,820
Babies born	35,417
Boys (%)	18,219 (51.9)
Girls (%)	16,868 (48.0)
Maternal age in years, mean (SD)	26.5 (5.3)
Maternal age distribution (%), n=35,084	
<20	13.1
21–35	81.2
>35	5.7
Body mass index (BMI), mean (SD), n=34,325	23.41 (4.2)
BMI distribution, (%)	
<18.5	5.7
18.5–25.0	64.9
>25.0	26.2
Parity, n=35,101	
0	19,962 (56.9)
1	12,425 (35.4)
≥2	2,714 (7.7)
Previous spontaneous abortions (%), n=35,043	
0	30,934 (88.3)
≥1	4,109 (11.7)
Education of mother in years (%), n=34,653	
≤11	13,046 (37.7)
>11	21,607 (62.3)
Paternal age, years, mean (SD)	29.5 (6.0)
Gestational age, GA, in weeks, mean (SD)	39.04 (2.3)
GA distribution, (%), n=33,694	
22–29	1.0
30–36	7.2
37–42	89.1
>42	2.7
Birth weight in g, mean (SD)	3,340 (553)
Multivitamins taken before pregnancy (%)	13.3
Multivitamins taken during pregnancy (%)	91.3
Folic acid intake before pregnancy (%)	8.5
Folic acid intake during pregnancy (%)	70.7
Smoking before pregnancy (%)	24.3
Smoking during pregnancy (%)	18.4
Alcohol abuse during pregnancy (%)	0.6
Drugs abuse during pregnancy (%)	0.5

SD, standard deviation.

We found 210 cases of major birth defects in the MCBR, compared to 195 in the MRCDR (Table 2). Of the 210 MCBR cases, 79 were not included in the MRCDR; conversely, 64 of the 195 cases in the MRCDR were not in the MCBR. In the combined registry, there were 274 cases of major birth defects. The updating of the MCBR dataset increased the overall prevalence of major birth defects from 55 to 77 per 10,000, which corresponds to an increase of 40%. A detailed comparison of the rates per 10,000 newborns of major birth defects in the MCBR and MRCDR is provided in Table 3. Both registries demonstrated the identical prevalence for seven out of the 21 major birth defects, namely; anencephaly, encephalocele, micro-anophthalmos, hypoplastic left heart syndrome, oesophageal atresia, exstrophy of the bladder and gastroschisis. For five major birth defects, the prevalences were comparable, namely: micro-anotia, ano-rectal atresia, renal agenesis and dysgenesis, diaphragmatic hernia and Down syndrome; and those for the remaining nine were more dissimilar (Table 3).

**Table 2.** Registration of major birth defects in Murmansk County 2006–2009.^a^

Type of defect	Cases recorded by both registries (1)	Cases recorded by MCBR only (2)	Cases recorded by MRCDR only (3)	Agreement^b^ (1)/[(1)+(2)+(3)]
Anencephaly; Q00	0	1	1	0%
Spina bifida; Q05	2	2	0	50%
Encephalocele; Q01	0	0	0	100%
Congenital hydrocephalus; Q03	10	2	7	53%
Anophthalmos, microphthalmos;Q11.0, Q11.2	1	0	0	100%
Anotia, microtia; Q16.0, Q17.2	3	0	1	75%
Transposition of great vessels; Q20.3	1	2	0	33%
Hypoplastic left heart syndrome; Q23.4	1	0	0	100%
Cleft palate; Q35	13	10	7	43%
Cleft lip with or without cleft palate; Q36.0, Q36.9, Q37	6	6	2	43%
Oesophageal atresia; Q39.0-Q39.4	4	2	2	50%
Ano-rectal atresia; Q42.0–Q42.3	4	1	0	80%
Renal agenesis or dysgenesis; Q60.1, Q60.4, Q60.6	3	2	1	50%
Hypospadias; Q54.0–Q54.3, Q54.8, Q54.9	41	38	12	45%
Epispadias; Q64.0	1	0	2	33%
Bladder exstrophy; Q64.1	1	0	0	100%
Limb reduction defects; Q71–Q73	11	3	20	32%
Diaphragmatic hernia; Q79.0	4	2	0	67%
Omphalocele; Q79.2	1	2	0	33%
Gastroschisis; Q79.3	5	0	0	100%
Down syndrome; Q90.0	19	6	9	56%
Total	131	79	64	47.8%

^a^ Major birth defects are those included in the mandatory MRCDR annual report. ^b^ Agreement refers to the percentage of total cases that are common between the two registries. MCBR, Murmansk County Birth Registry; MRCDR, Murmansk Regional Congenital Defects Registry.

**Table 3.** Registration of major birth defects^a^; Murmansk County 2006–2009 (n=35,417).

	MCBR		MRCDR		New combined registry	
Type of birth defect; ICD-10 code	n	rate^b^	n	rate^b^	n	rate^b^
Anencephaly; Q00	1	0.3	1	0.3	2	0.6
Spina bifida; Q05	4	1.1	2	0.6	4	1.1
Encephalocele; Q01	0	0	0	0	0	0
Congenital hydrocephalus; Q03	12	3.4	17	4.8	19	5.4
Anophthalmos, microphthalmos; Q11.0, Q11.2	1	0.3	1	0.3	1	0.3
Anotia, microtia; Q16.0, Q17.2	3	0.8	4	1.1	4	1.1
Transposition of great vessels; Q20.3	3	0.8	1	0.3	3	0.8
Hypoplastic left heart syndrome; Q23.4	1	0.3	1	0.3	1	0.3
Cleft palate; Q35	23	6.5	20	5.6	30	8.5
Cleft lip with or without cleft palate; Q36.0, Q36.9, Q37	12	3.4	8	2.3	14	4.0
Oesophageal atresia; Q39.0–Q39.4	6	1.7	6	1.7	8	2.3
Ano-rectal atresia; Q42.0–Q42.3	5	1.4	4	1.1	5	1.4
Renal agenesis or dysgenesis; Q60.1, Q60.4, Q60.6	5	1.4	4	1.1	6	1.7
Hypospadias; Q54.0–Q54.3, Q54.8, Q54.9	79	22.3	53	15	91	25.7
Epispadias; Q64.0	1	0.3	3	0.8	3	0.8
Bladder exstrophy; Q64.1	1	0.3	1	0.3	1	0.3
Limb reduction defects; Q71–Q73	14	4	31	8.8	34	9.6
Diaphragmatic hernia; Q79.0	6	1.7	4	1.1	6	1.7
Omphalocele; Q79.2	3	0.8	1	0.3	3	0.8
Gastroschisis; Q79.3	5	1.4	5	1.4	5	1.4
Down syndrome; Q90.0	25	7	28	7.9	34	9.6
Total	210	60	195	55	274	77

^a^ Major birth defects are those included in the mandatory MRCDR annual report. ^b^ Rate per 10,000 newborns. MCBR, Murmansk County Birth Registry; MRCDR, Murmansk Regional Congenital Defects Registry

To the extent possible, the prevalence of major birth defects in Murmansk County were also compared with those in Arkhangelsk County [18–21] and in Norway [22–25] for the years 2006–2009 (in rates per 10,000; Table 4). We decided to use the Norwegian data representing the whole country instead of different regions because of the uniform distribution of birth defects across Norway. We removed abortions data before 22 weeks of gestation from the Norwegian dataset to reflect the absence of such data in the Russian dataset. Compared with Murmansk County, Arkhangelsk County demonstrated a higher prevalence of birth defects of the nervous system, namely: anencephaly, spina bifida and encephalocele, whereas those from Norway were more comparable. The prevalence of oesophageal atresia and ano-rectal atresia were almost identical in the three areas. In Murmansk County, the prevalence of limb reduction defects and hypospadias was higher than in Arkhangelsk County and Norway. Among the three locations, Murmansk County had the highest prevalence of cleft palate and the lowest prevalence of cleft palate and lip.

**Table 4.** National and international comparisons of birth defects for 2006–2009, rate per 10,000 newborns (includes livebirths, stillbirths and terminations at 22 weeks and beyond).

	Arkhangelsk County^a^ (n=58,141)		Murmansk County “Combined registry” (n=35,417)		Norway^b^ (n=243,231)	
Type of birth defect	n	rate (95% CI)	n	rate (95% CI)	n	rate (95% CI)
Anencephaly; Q00	40	6.9 (5–9)	2	0.6 (0–1)	9	0.4 (0–1)
Spina bifida; Q05	55	9.5 (7–12)	4	1.1 (0–2)	46	1.9 (1–2)
Encephalocele; Q01	11	1.9 (1–3)	0	0	10	0.4 (0–1)
Congenital hydrocephalus; Q03	27	4.6 (3–6)	19	5.4 (3–8)	73	3.0 (2–4)
Anophthalmos, microphthalmos; Q11.0, Q11.2	2	0.3 (0–1)	1	0.3 (0–1)	—	—
Anotia, microtia; Q16.0, Q17.2	3	0.5 (0–2)	4	1.1 (0–2)	10	0.4 (0–1)
Transposition of great vessels; Q20.3	16	2.8 (2–5)	3	0.8 (0–2)	102	4.2 (3–5)
Hypoplastic left heart syndrome; Q23.4	18	3.1 (2–5)	1	0.3 (0–1)	46	1.9 (1–2)
Cleft palate; Q35	14	2.4 (1–4)	30	8.5 (5–12)	164	6.7 (6–8)
Cleft lip with or without cleft palate; Q36.0, Q36.9, Q37	30	5.2 (3–7)	14	4.0 (2–6)	291	12.0 (11–13)
Oesophageal atresia; Q39.0–Q39.4	14	2.4 (1–4)	8	2.3 (1–4)	58	2.4 (2–3)
Ano-rectal atresia; Q42.0–Q42.3	9	1.5 (1–3)	5	1.4 (0–3)	60	2.5 (2–3)
Renal agenesis or dysgenesis; Q60.1, Q60.4, Q60.6	0	0	6	1.7 (0–3)	12	0.5 (0–1)
Hypospadias; Q54.0–Q54.3, Q54.8, Q54.9	24	4.1 (2–6)	91	25.7 (2–31)	317	13.0 (12–14)
Epispadias; Q64.0	0	0	3	0.8 (0–2)	—	—
Bladder exstrophy; Q64.1	2	0.3 (0–1)	1	0.3 (0–1)	—	—
Limb reduction defects; Q71–Q73	10	1.7 (1–3)	34	9.6 (6–13)	76	3.1 (2–4)
Diaphragmatic hernia; Q79.0	7	1.2 (0–2)	6	1.7 (0–3)	50	2.1 (1–3)
Omphalocele; Q79.2	23	4.0 (2–6)	3	0.8 (0–2)	30	1.2 (1–2)
Gastroschisis; Q79.3	17	2.9 (2–4)	5	1.4 (0–3)	79	3.2 (3–4)
Down syndrome; Q90.0	68	11.7 (9–14)	34	9.6 (6–13)	309	12.7 (11–14)
Total	390	67 (60–74)	274	77 (68–86)	1742	72 (68–75)

^a^ Data from Arkhangelsk Regional Congenital Defects Registry. ^b^ Data from Norwegian Birth Registry. CI, confidence interval.

## Discussion

To the authors’ knowledge, this is the first time that a birth registry and a birth defect registry have been combined in Russia to determine the prevalence of birth defects. We found that 79 of the 210 cases of major birth defects (i.e. for the 21 birth defects included in the mandatory MRCDR annual report) registered in the MCBR were not included in the MRCDR. We, therefore, demonstrated a 40% increase in the overall prevalence of major birth defects after combining the two registries.

Before 2006, there were no adequate mechanisms to estimate the completeness of the MRCDR and, consequently, there are no published reports regarding its quality. When comparing the MCBR and the MRCDR, we found that the former had better case ascertainment. The most likely explanation for this is that the registration routines were better in the MCBR, such as regular quality controls, having only one person responsible for registration in each maternity hospital, strict delivery of birth registry forms to the central office using courier services and in general having fewer individuals involved in the data chain. In contrast, the MRCDR draws upon all health institutions and, thereby, involves more people and fewer quality control routines. Furthermore, it covers the neonatal period and includes diagnoses for the child to 16 years of age.

Our study is, therefore, an example of how useful registry linkage can be. It revealed significant under-reporting of some major birth defects in Murmansk County, which led to under-reporting of the overall rate of birth defects in this region. Our findings provide decision-makers with insight about a need for suitable and routine quality control measures to guarantee the quality of public health statistics.

Certain population characteristics may influence the prevalence of birth defects and, therefore, it is important to compare them for the same period to those of neighbouring jurisdictions such as Arkhangelsk County and Norway. The average age of mothers (at the time of delivery) in Murmansk County was 26.5 years, which is lower than that in Norway, where it was 29.6 years during study period [10]. The proportion of mothers over 35 years of age at the time of delivery in Murmansk County was 5.7%, while it was 16.7% in Norway [10]. Advanced maternal age is significantly associated with an increased risk for a variety of birth defects [26], including those of the heart and Down syndrome [27]. In our study, the prevalence of these two defects was lower in Murmansk County than in Norway, which likely reflects the lower average maternal age observed in Murmansk County.

Folic acid supplementation reduces the risks of spina bifida and some ano-rectal atresia, as well as of selected orofacial clefts in high doses [28–31]. The use of multivitamins and folic acid during pregnancy in Murmansk County are attributable to existing programmes of the Ministry of Health Care in the region. Furthermore, these supplements are available free of charge for pregnant women. However, the pertinent studies also show that folic acid intake is most effective in preventing birth defects when taken prior to conception. In our study, only 8.5% of mothers in Murmansk County took folic acid before pregnancy, while in Norway this percentage was 27.4%. Even with higher folic acid intake by Norwegian mothers, the prevalence of neural tube defects in Murmansk County was slightly lower, although this was not statistically significant. A higher prevalence of birth defects of the nervous system (including anencephaly, spina bifida, encephalocele and hydrocephalus) occurred in Arkhangelsk County relative to Murmansk County and Norway (for which they were comparable [32]). Poverty and food insecurity during the study period were cited as potential contributing factors (including low folic acid intake before and during pregnancy). Since Arkhangelsk County is larger and more rural compared to Murmansk County, a lower availability of regular ultrasound screening might have led to later diagnoses of birth defects (i.e. after 22 weeks of gestation).

More than 90% of pregnant women in Murmansk County undergo ultrasound examinations at least three times during their pregnancy, with the first one usually at about 12 (12.4 weeks on average) weeks of gestation, as required by Federal Order № 572 from the Ministry of Health Care of the Russian Federation [33]. According to unpublished data from the Ministry of Health Care of Murmansk Oblast, thorough ultrasound observations help detect around 100 each of major and minor birth defects every year and about 50% of these women decide to continue the pregnancy, despite the presence of birth defects.

Maternal smoking is also associated with increased risk of birth defects, specifically missing or malformed limbs and facial disorders [34]. In Norway, smoking during the first trimester was associated with an increased risk of cleft lip, with or without cleft palate [35]. We observed that the prevalence of cleft lip was lower in Murmansk County compared to both Arkhangelsk County and Norway. This observation appears to be inconsistent with the high percentage of women in Murmansk County who smoked both before and during pregnancy (respectively, 24.3% and 18.4%). While the prevalence of cleft palate was the highest in Murmansk County, cleft lip with or without cleft palate was the lowest. Ethnic and racial differences, misclassification, wrong coding and/or possible under-reporting of cleft lip in Murmansk Oblast may well have contributed to this discrepancy compared to Norway.

Generally speaking and based on the combined Murmansk County registries, the overall prevalence of major birth defects of 77 per 10,000 compared well with the 67 in Arkhangelsk County and 72 in Norway. Without linking the two registries, Murmansk County would have exhibited the lowest prevalence (55 per 10,000).

For oesophageal atresia and ano-rectal atresia, the prevalence ranged from 1.5–2.5 per 10,000 in all three locations and was, thus, too rare to allow adequate comparisons. These two defects are easily recognisable at birth and require urgent surgical treatment.

In Murmansk County, the prevalence of limb reduction defects was unexpectedly high (9.9), while in Arkhangelsk County it was 1.7 and in Norway 3.1 per 10,000. Detailed analysis revealed that 10 such cases were recorded with the same ICD-10 code and were all diagnosed in the military town of Gadzhiyevo, which has a population of about 11,000 and around 250 annual deliveries [16]. All 10 cases recorded in the MRCDR database were reported for the same children’s polyclinic, where a single doctor was responsible for regular infant check-ups. The description of all 10 of these cases in the MRCDR database was “developmental hypoplasia of the hip”, but the code used was Q71. Incorrect coding here is partly responsible for the overall high prevalence of limb reduction defects in the County. Clearly, this needs further confirmation and follow-up. The prevalence of hypospadias was high in Murmansk County (25.4) and Norway (13.0), but low in Arkhangelsk County (4.1). Even though 70% of hypospadias cases in Murmansk County were identified during the perinatal period, the remainder occurred between the neonatal and infant periods. Our detailed analysis revealed an even distribution throughout Murmansk County in relation to population size. This suggests that no systematic error was present, but this requires closer examination. Another possibility is that mild forms of hypospadias in Arkhangelsk County were not registered.

### Strengths of the study

We describe a successful linkage of records from a birth defects registry with those of a medical birth registry, based on original hospital data, hospital ID number and the last name of the mother. The established satisfactory quality of the MCBR constitutes a strength [10]. Although federal law dictates that neonatal data be collected from week 22 of gestation on, the MRCDR does not contain data on infants below 970 grams (which equates to approximately 27–28 weeks). This is a remnant of the earlier Russian system before 2012 that considered that termination of a pregnancy at 22–27 weeks was a spontaneous/induced abortion, not a pre-term delivery. Potential under-reporting of birth defects might have occurred because women at 22–27 weeks of pregnancy gave birth in a hospital gynaecology department. Fortunately, the MCBR covered this period.

### Limitations of the study

The dependence on the experience of the medical doctors to detect and correctly diagnose birth defects, especially in remote areas, may cause systematic errors such as under-reporting, over-reporting and misclassification. Another limitation is that elective abortions due to birth defects (<22 weeks of gestation) were not included in the Murmansk County and Arkhangelsk County registries. This hindered our attempts to acquire more accurate prevalence estimates. Moreover, differences in pre-natal diagnostics algorithms of birth defects and early terminations may also have contributed to rate differences in the regions compared. Information from the Murmansk Genetics Centre (MGC) could potentially include pregnancy terminations due to birth defects diagnosed pre-natally by the MGC. Although these data were available, they were not included in the MCBR. Another limitation is that some selected defects were so rare (as might be expected) that comparisons of rates lacked statistical power.

## Conclusions

A number of studies have indicated substantial under-reporting of birth defects based on statutory notifications of births compared with hospital records and this was the case in Murmansk County. A surveillance system solely based on notifications of births is not advocated [36]. Under-reporting of prevalence like that found in our study hides the extent to which birth defects affect a population. When such information is part of the planning or evaluation of prevention strategies it can lead to erroneous conclusions about the effectiveness of a programme and can influence health policies and the allocation of resources [37]. Our study demonstrates that birth registry data can serve to improve existing surveillance data, increases case ascertainment and reduces the effects of possible under-reporting by physicians. This is an effective approach to enhance birth defects surveillance.

## Availability of supporting data

The MCBR and MRCDR databases have restricted access due to privacy issues and patient confidentiality. Permission to use data requires the submission of an application. For access to the MCBR data, permission of both the Ministry of Health Care of the Murmansk Region and the UiT Arctic University of Norway is required. For the MRCDR data, access requires permission of the Ministry of Health Care of the Murmansk Region.
